# Fetal Magnetocardiography Using Optically Pumped Magnetometers: A Literature Review

**DOI:** 10.3390/bios16090487

**Published:** 2026-09-02

**Authors:** Rok Hren, Urban Marhl, Tamás Dóczi, Erika Országh, Vojko Jazbinšek, Tilmann Sander

**Affiliations:** 1 Syreon Research Institute, 1142 Budapest, Hungary; 2 Faculty of Mathematics and Physics, University of Ljubljana, 1000 Ljubljana, Slovenia; 3 Institute of Mathematics, Physics and Mechanics, 1000 Ljubljana, Slovenia; 4 School of Engineering and Management, University of Nova Gorica, 5000 Nova Gorica, Slovenia; 5 Faculty of Natural Sciences and Mathematics, University of Maribor, 2000 Maribor, Slovenia; 6 Physikalisch-Technische Bundesanstalt, 10587 Berlin, Germany; tilmann.sander-thoemmes@ptb.de

**Keywords:** fetal magnetocardiography, optically pumped magnetometers, quantum sensors, fetal arrhythmia, fetal electrocardiology, prenatal diagnosis, cardiac electrophysiology, SQUID, biomagnetism, magnetic sensing

## Abstract

Fetal magnetocardiography (fMCG) provides direct non-invasive assessment of fetal cardiac electrophysiology, enabling detailed evaluation of cardiac rhythm, conduction, and repolarization. However, the clinical adoption of conventional fMCG has been limited by its reliance on superconducting quantum interference device (SQUID) systems, which require cryogenic cooling and specialized infrastructure. Optically pumped magnetometers (OPMs) have emerged as a promising cryogen-free alternative with the potential to broaden access to fetal electrophysiological assessment. This review summarizes the technological evolution and early clinical evaluation of OPM-based fMCG through an analysis of original in vivo human studies published up to June 2026. Twelve eligible studies were identified and synthesized narratively. Advances in sensor design, magnetic shielding, acquisition strategies, and signal-processing algorithms have enabled SQUID-comparable signal quality and cardiac interval measurements while substantially reducing cryogenic and infrastructure requirements. OPM-fMCG has demonstrated the potential to assess fetal cardiac time intervals, heart rate variability, fetal movement, and clinically important arrhythmias, including congenital long QT syndrome, atrioventricular block, and supraventricular and ventricular tachyarrhythmias. However, the available evidence remains dominated by small, single-centre studies, with relatively few fetuses affected by clinically significant arrhythmias. Prospective multicenter clinical validation, protocol standardization, independent replication, and regulatory evaluation are therefore required before OPM-fMCG can be integrated into routine diagnostic pathways for pregnancies requiring advanced fetal electrophysiological assessment.

## 1. Introduction

Current fetal cardiac assessment relies primarily on fetal echocardiography, which excels at evaluating cardiac anatomy and mechanical function but has important limitations in assessing cardiac electrophysiology [1]. Although recent studies have found an association between prolonged isovolumic relaxation time and long QT syndrome (LQTS) as well as other inherited arrhythmia syndromes (IASs) [2,3], echocardiography can not reliably measure cardiac conduction intervals (e.g., PR, QRS, QT), identify repolarization abnormalities, or diagnose several clinically important arrhythmias, including LQTS, Wolff-Parkinson-White syndrome, bundle branch block, or Torsades de Pointes (TdP) [2,4]. As a result, potentially life-threatening fetal arrhythmias may remain undetected until birth or fetal demise.

Fetal magnetocardiography (fMCG), first recorded in 1974 [5], is a non-invasive technique for measuring the magnetic fields generated by the electrical activity of the fetal heart. Unlike fetal electrocardiography (ECG) [6,7], magnetic signals are minimally affected by the electrical conductivity of maternal tissues and vernix caseosa [8,9], enabling reliable electrophysiological assessment throughout gestation. Similar to postnatal ECG, fMCG provides precise measurements of cardiac time intervals (P-wave duration and PR, RR, QRS, QT, and QTc intervals), waveform morphology, and complex cardiac rhythm patterns. Reflecting these capabilities, the American Heart Association Scientific Statement assigns fMCG a Class IIa recommendation for evaluating cardiac conduction and rhythm in fetuses with known or suspected conduction abnormalities, noting that no other available technique provides such direct and precise electrophysiological assessment [10].

Given that IASs are estimated to account for 3–9% of unexplained stillbirths, approximately 10% of sudden infant death syndrome, and up to 30% of sudden cardiac deaths in young individuals [2], there is a clear need for accessible prenatal electrophysiological assessment. Among its clinical applications, fMCG has demonstrated particular value for the prenatal diagnosis of LQTS [11,12,13,14,15], including de novo forms that are especially difficult to recognize and are associated with a high risk of fetal mortality [16]. In a retrospective study of 144 high-risk pregnancies referred for fetal arrhythmias or IASs, Wacker-Gussmann et al. [17] showed that fMCG provided additional or revised diagnostic findings in 81% of cases compared with referral echocardiography, resulting in critical diagnostic changes in 56% and major changes in clinical management in 24% of pregnancies, largely through improved detection of LQTS, TdP, and complex conduction abnormalities. Due to its clinical value, a superconducting quantum interference device (SQUID)-based fMCG system received U.S. Food and Drug Administration (FDA) 510(k) clearance in 2016 [2,18].

Despite its proven clinical utility, widespread adoption of fMCG has been limited by dependence on SQUID-based instrumentation. Although SQUID systems provide exceptional magnetic sensitivity, they require cryogenic cooling with liquid helium, large magnetically shielded rooms (MSRs), and specialized maintenance, resulting in high acquisition and operating costs that restrict their availability to a small number of specialized centers worldwide.

Optically pumped magnetometers (OPMs) [19,20,21,22,23,24,25,26,27,28,29] offer a promising alternative by achieving magnetic sensitivities approaching those of SQUIDs while operating at room temperature. Their compact size permits flexible sensor placement closer to the fetal heart, partially compensating for their higher intrinsic noise, while eliminating the need for cryogenic infrastructure. OPM-based systems have already reported performance comparable to conventional SQUID systems in adult magnetoencephalography (MEG) and magnetocardiography, while providing greater flexibility, lower infrastructure requirements, and improved patient comfort [30,31]. These characteristics make OPMs particularly well suited for fMCG, where adaptable sensor geometries can better conform to the maternal abdomen and improve signal quality, while compact shielding and cryogen-free operation may ultimately facilitate deployment beyond the small number of highly specialized SQUID laboratories. Nevertheless, current OPM-fMCG systems continue to require specialized magnetic shielding, field-control strategies, and substantial technical expertise, and should not yet be regarded as point-of-care or plug-and-play technologies. A recent narrative review [2] has summarized fMCG systems, including the emergence of OPMs, and emphasized their potential clinical applications. However, the expanding body of OPM-fMCG research has not previously been evaluated in a focused review using a structured literature search and transparent narrative evidence synthesis focused specifically on the translational development of OPM technology. The present review addresses this gap by critically evaluating all available in vivo clinical studies of OPM-fMCG, examining its technological evolution, methodological development, diagnostic performance, and clinical applications, while identifying the remaining scientific, technical, regulatory, and implementation challenges that must be overcome before OPM-fMCG can become a standardized tool for routine fetal cardiac assessment.

## 2. Materials and Methods

A comprehensive literature search was conducted on 14 June 2026 using PubMed, Embase and Scopus, with no limitations on publication date while database-specific publication-type filters were applied as described in the Appendix A. The scope of the review and article-selection criteria were defined in advance and are also detailed in the Appendix A. Title and abstract screening, full-text assessment, and data extraction were performed by two reviewers, with disagreements resolved by consensus. Original in vivo human studies evaluating fMCG performed with OPMs in pregnant participants were eligible, while animal, phantom, ex vivo, purely technical development studies without original in vivo pregnant-human fetal cardiac outcomes, or purely methodological investigations were excluded as were reviews. Duplicate records were removed both within and across databases. When identical work was published first in conference proceedings and later in a peer-reviewed journal, the proceedings version was treated as a secondary report and discarded. Records that did not meet any exclusion criteria at the full-text screening stage were included. A more detailed description of the methodology, including PICOS, search strings, and inclusion and exclusion criteria during title/abstract and full-text screening, is reported in the Appendix A. To ensure that no recently published or poorly indexed studies were missed, a brief, targeted supplementary search was performed in specialist journals, Google Scholar and reference lists.

## 3. Results

**Figure 1:**
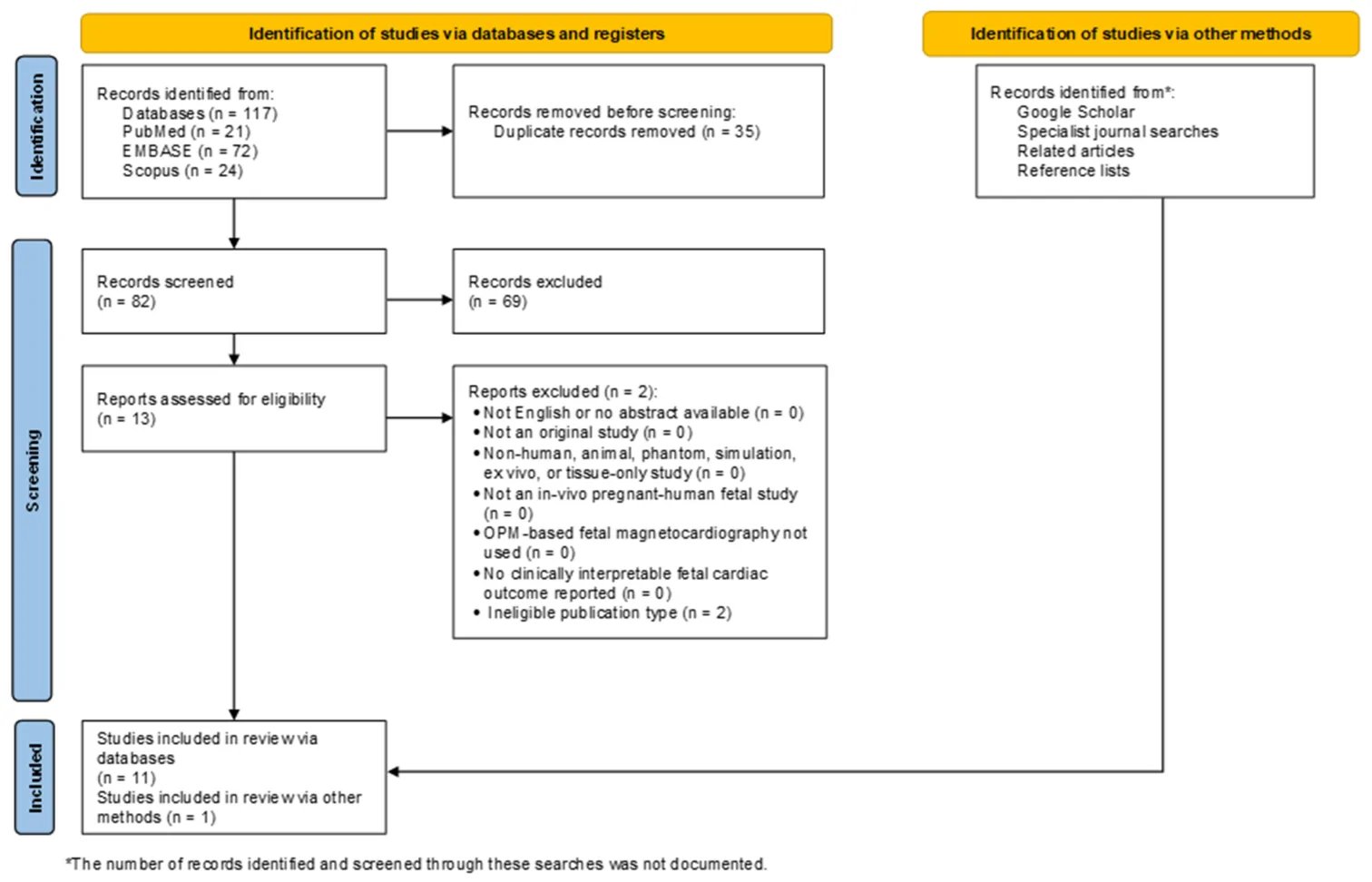
PICOS flow chart of comprehensive review.

A total of 117 records were identified through database searches (PubMed: 21, Embase: 72, Scopus: 24). After removal of 35 duplicates using Deduplicator [32], 82 records underwent title and abstract screening, of which 69 were excluded. Thirteen articles were assessed for full-text eligibility, and eleven met the inclusion criteria. The supplementary search identified one additional eligible study, resulting in a total of 12 studies included in this review [33,34,35,36,37,38,39,40,41,42,43,44]. The study selection process is summarized in the PICOS flow diagram (Figure 1). The included studies were categorized into early proof-of-concept investigations and three clinical research programs (clinical-study “clusters”) conducted in Madison (WI, USA), Little Rock (AR, USA), and Munich (Germany). Clinical studies were grouped by research program to preserve the developmental continuity of each platform while enabling comparison of complementary approaches across programs. For the purposes of narrative synthesis, four studies were considered key translational steps because they represented first clinical evaluation (Section 3.2.1), compact-system deployment (Section 3.2.2), stand-alone system integration (Section 3.3.4), and evaluation of gestational-age-specific reference intervals (Section 3.4.2). Given the predominance of feasibility and technical-clinical studies with heterogeneous designs, methodological quality was assessed narratively rather than through a single formal appraisal tool. In addition, since several publications originated from the same centers and sequentially evaluated related platforms, overlap between participant cohorts could not always be excluded. The characteristics of the included studies are summarized in Table 1, while a comparison of their experimental setups and signal processing pipelines is provided in Table 2.

### 3.1. Early Studies

Together, these two proof-of-concept studies [33,34] established the technical feasibility of OPM-fMCG, indicating that cryogen-free sensors could achieve signal quality approaching conventional SQUID systems while introducing flexible sensor geometries.

#### 3.1.1. Proof-of-Concept OPM-Based Fetal Magnetocardiography

Wyllie et al. [33] presented the first fMCG using an array of spin-exchange relaxation-free (SERF) OPMs. The prototype consisted of a four-channel array arranged in a 7-cm square geometry, with each sensor employing a localized optical pumping region surrounded by a larger pump-free detection volume to minimize pump-induced relaxation and AC Stark effects. Active magnetic field compensation was implemented by feeding the output of one sensor back to a field coil, creating a gradiometric configuration that suppressed uniform environmental interference by approximately 40 dB while preserving fetal cardiac signals. Despite the small number of sensors, the system achieved real-time recordings from a healthy fetus at 31 weeks’ gestation and found agreement with a commercial 21-channel SQUID system for cardiac intervals (RR, PR, QRS, and QT), while also clearly identifying the fetal QRS complex and, importantly, the fetal P wave.

#### 3.1.2. Conformal Multichannel OPM Array

Alem et al. [34] introduced a large-scale, flexible conformal array comprising 25 microfabricated SERF optically pumped magnetometers (μOPMs) specifically designed for obstetric application. In contrast to the earlier four-sensor prototype, the magnetometers were miniaturized to approximately 1 cm^3^, integrated into flexible belt-shaped holders, and positioned over the maternal abdomen and chest to conform to individual anatomy throughout pregnancy. Optical fibers connected the room-temperature sensors to external electronics, allowing for sensor placement within 5 mm of the skin. Signal extraction combined orthogonal projection (OP) and independent component analysis (ICA) to suppress maternal cardiac activity and isolate fetal signals. Although only 16 of the 25 sensors were retained for analysis (9 channels exhibiting excessive fluctuations were excluded), the prototype produced recordings from a healthy fetus at 32 weeks’ gestation with signal quality approaching that of SQUID recordings. However, the experiment was conducted in the highly shielded seven-layer BMSR II, and the authors identified sensor-to-sensor variability, fiber-associated noise, and the need for reliable operation in less extensively shielded environments as important remaining engineering challenges.

### 3.2. Clinical Studies—Madison Program

The Madison program established clinical evidence supporting OPM-fMCG by demonstrating diagnostic performance comparable to SQUID systems and subsequently developing a compact platform suitable for clinical evaluation.

#### 3.2.1. Clinical Evaluation Against SQUID Systems

Batie et al. [35] reported the first direct clinical comparison between SQUID- and OPM-based fMCG in the 2-shell MSR. Using a configurable array of three to eight QuSpin zero-field OPMs, the investigators showed that increasing the sensor count from fewer than six to eight sensors, together with improved acquisition electronics, increased the signal-to-noise ratio (SNR) to 74–104% of that achieved by the FDA-cleared 21-channel SQUID system. These findings indicated that cryogen-free OPM technology can provide diagnostic-quality fetal cardiac recordings, with performance strongly influenced by sensor count and system optimization.

Clinically, the study evaluated 15 pregnancies, including 7 with suspected or confirmed fetal arrhythmias. The OPM system successfully identified congenital LQTS and severe QT prolongation, ventricular bigeminy with intermittent ventricular tachycardia, low atrial rhythm associated with heterotaxy, and atrial and ventricular ectopy. OPM-fMCG accurately characterized both rhythm disturbances and ventricular repolarization abnormalities, reproducing the diagnostic information obtained with SQUID while supporting prenatal diagnosis and clinical management.

#### 3.2.2. Compact OPM System for Clinical Fetal Magnetocardiography

Strand et al. [36] introduced a compact OPM-fMCG system capable of providing clinically viable recordings, representing an important step toward replacing conventional SQUID technology. The platform incorporated 10-sensor (20-channel) dual-axis QuSpin OPMs mounted in a custom 3D-printed array and operated within a compact, person-sized cylindrical magnetic shield, thereby eliminating the need for both cryogenic cooling and large MSRs. Although the open-ended shield was associated with increased environmental magnetic noise, this limitation was effectively mitigated by positioning participants in the prone position, which reduced the sensor-to-fetus distance and increased signal amplitude by an average factor of 2.63. Consequently, the OPM system achieved SNRs comparable to those of the FDA-cleared 21-channel SQUID instrument. The systems demonstrated high concordance for PR, QTc, and RR intervals, with coefficients of 0.98, 0.97, and 0.95, respectively, whereas agreement for QRS duration was lower at 0.74.

The investigators compared OPM- and SQUID-based fMCG recordings in 24 pregnancies, including 16 fetuses referred for suspected or confirmed arrhythmias, and reported comparable diagnostic findings between the two technologies. OPM-fMCG accurately identified inherited repolarization disorders (including LQTS, QT prolongation, T-wave alternans, and TdP) as well as atrial and ventricular arrhythmias, including atrial flutter, atrial ectopic tachycardia, blocked atrial bigeminy, atrioventricular block, ventricular tachycardia, and ectopic beats. In several cases, OPM-fMCG provided more precise electrophysiological characterization than fetal echocardiography by distinguishing the mechanisms underlying tachyarrhythmias and bradyarrhythmias and by identifying ventricular repolarization abnormalities.

### 3.3. Clinical Studies—Little Rock Program

The Little Rock program advanced OPM-fMCG from adaptable sensor arrays to integrated clinical systems. Their work introduced flexible conformal arrays (Section 3.3.1), evaluated fetal heart rate variability (FHRV) in a larger cohort (Section 3.3.2), improved maternal ergonomics through bed-based system design (Section 3.3.3 and Section 3.3.4), extended OPM-fMCG to fetal movement assessment (Section 3.3.5), and identified the principal technical and physiological determinants of recording quality (Section 3.3.6).

#### 3.3.1. Pilot Evaluation of Adaptable OPM Arrays

Escalona-Vargas et al. [37] developed a flexible 14-channel OPM array capable of conforming to the maternal abdomen while acquiring high-quality fetal cardiac signals in multiple maternal positions. Using custom 3D-printed sensor holders integrated into flexible belts, the system demonstrated one of the principal advantages of OPM technology over rigid SQUID arrays by permitting recordings in forward-leaning, backward-leaning, and prone positions. Although evaluated in only three uncomplicated pregnancies, the pilot study successfully measured clinically relevant FHRV parameters, including fetal heart rate, RR intervals, RMSSD, and SDNN, with values comparable to those previously reported using SQUID-based systems. As this pilot evaluated the same adaptable-array concept later examined in a larger cohort by Escalona-Vargas et al. [38], overlap between the participant samples could not be excluded.

#### 3.3.2. Clinical Evaluation of Adaptable OPM Arrays for Fetal Heart Rate Variability Assessment

Building on this pilot study, Escalona-Vargas et al. [38] evaluated the same adaptable 14-channel concept in 24 healthy pregnancies between 28 and 38 weeks’ gestation. Fetal cardiac activity was successfully detected in 88% of participants (participant-level overall detection rate), with detection rates improving after 32 weeks. FHRV estimates broadly overlapped with those from gestational-age-matched historical SQUID recordings, although short-term variability measures showed discrepancies attributed partly to missed beats and movement artefacts. Although limited to uncomplicated pregnancies, the study provided a large-scale clinical evaluation of flexible OPM arrays for quantitative fetal physiological assessment.

#### 3.3.3. Prototype Bed-Based OPM System

Escalona-Vargas et al. [39] next focused on improving maternal comfort by introducing a prototype bed-based OPM-fMCG system in which a 14-sensor (28-channel) array was mounted beneath a customized prone-position examination bed. By reducing the sensor-to-fetus distance, fMCG was detected in 13 of 15 participants, with sufficient signal quality for continuous RR-interval analysis in 11, while FHRV measurements remained in agreement with established SQUID-based values.

#### 3.3.4. Stand-Alone Bed-Based OPM System

Escalona-Vargas et al. [40] subsequently reported the first fully integrated stand-alone OPM-fMCG platform, combining a compact cylindrical magnetic shield with a customized examination bed and a 14-sensor (28-channel) dual-axis array. In 72 paired (back-to-back not simultaneous) OPM-SQUID recordings obtained from 22 pregnancies, the fMCG system achieved a fetal recording/dataset-level detection rate of 91.7%, while cardiac time intervals (P-wave duration and PR, QRS, and QT intervals) and FHRV closely matched those obtained using the reference SQUID system.

#### 3.3.5. OPM-Based Fetal Movement Assessment

Building on earlier SQUID-fMCG work of Lutter and Wakai et al. [56], which demonstrated fetal movement assessment from biomagnetic recordings, Escalona-Vargas et al. [41] extended this capability to the stand-alone OPM-fMCG. Integration of precise sensor localization with automated signal processing methods enabled the generation of fetal movement actograms from recordings obtained in four uncomplicated pregnancies, providing preliminary evidence that the same OPM-fMCG acquisition may support concurrent electrophysiological and fetal movement assessment.

#### 3.3.6. Factors Influencing OPM-fMCG Signal Quality

Ramirez et al. [42] systematically investigated the factors influencing OPM-fMCG signal quality using the stand-alone platform in 107 longitudinal recordings obtained from 32 pregnancies. Detection rates reached 100% for the QRS complex, 92% for the P wave and PR interval, and 83% for the QT interval, whereas the T wave remained the most challenging waveform component to detect. Gestational age was positively associated with recording quality, while sensor-to-fetus distance emerged as the strongest determinant of SNR. After adjustment for gestational age and fetal-heart distance, maternal body mass index and placental position were not independent predictors of signal quality. These findings provide practical guidance for optimizing sensor placement and acquisition protocols. As this study evaluated the same stand-alone platform and a similar longitudinal cohort as the earlier investigation by Escalona-Vargas et al. [40], overlap between participant samples is likely but could not be confirmed.

### 3.4. Clinical Studies—Munich Program

The Munich program focused on implementing hospital-based OPM-fMCG [43] and establishing gestational-age-specific reference intervals [44].

#### 3.4.1. Hospital-Based Implementation of OPM-fMCG

Wurm et al. [43] reported the first European hospital-based implementation of OPM-fMCG by integrating the technology into a pediatric cardiology department. The system consisted of an 8-sensor (16-channel) OPM array housed within a compact, person-sized three-layer cylindrical mu-metal magnetic shield, eliminating the need for a conventional walk-in MSR. Pregnant women were examined in the prone position, allowing the maternal abdomen to be positioned close to the sensor array, thereby maximizing fetal signal amplitude. Ten-minute recordings from seven uncomplicated pregnancies between 26 and 36 weeks’ gestation were processed using ICA and matched filtering to suppress maternal cardiac interference and generate high-quality averaged fetal waveforms. The resulting measurements of fetal cardiac intervals (PR, QRS, and QTc) closely agreed with previously published SQUID- and OPM-based reference values, demonstrating that diagnostic-quality fMCG recordings can be achieved using a compact, cryogen-free system within a hospital setting. Residual environmental magnetic interference and vibration artifacts remained the principal limitations to single-beat signal quality, indicating priorities for further system optimization.

#### 3.4.2. Establishment of Gestational-Age-Specific Reference Intervals

Wacker-Gussmann et al. [44] established the first gestational age-related OPM-fMCG prediction intervals in a comparatively large healthy cohort, representing an important step in the clinical translation of the technology. The study enrolled 57 healthy singleton pregnancies, of which 5 recordings were excluded because of a low SNR or magnetic artefacts. Recordings were acquired using a 16-sensor dual-axis QuSpin OPM array within a compact person-sized cylindrical magnetic shield. Active magnetic field compensation, combined with independent component analysis, digital filtering, fetal actography, and beat averaging, enabled high-quality recordings from which cardiac time intervals (P-wave duration and PR, QRS, QT, QTc, and RR intervals) were measured. The resulting interval values were consistent with previously published SQUID-derived reference data.

Cardiac maturation was characterized by progressive increases in P-wave duration, PR interval, and QRS duration with advancing gestation, whereas QT and QTc intervals remained relatively stable. Comparison with postnatal ECGs obtained from 11 infants showed the expected physiological changes after birth, including increases in P-wave duration, QRS duration, and QTc, while PR and QT intervals remained largely unchanged.

## 4. Discussion

**Figure 2:**
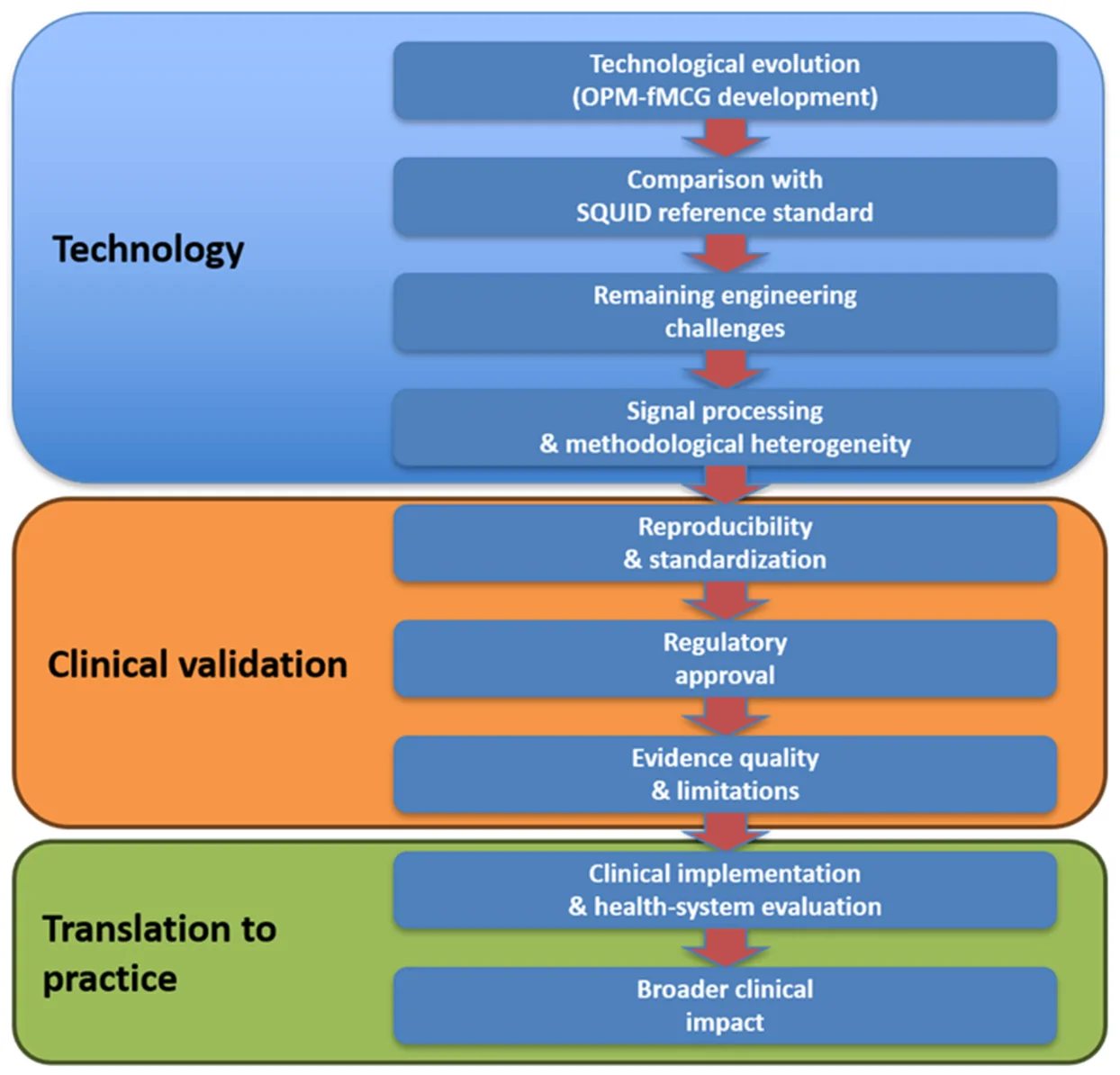
The translational pathway for the clinical implementation of OPM-based fetal magnetocardiography (OPM-fMCG). The framework illustrates the key stages discussed in this review, progressing from technological development and validation through methodological standardization, reproducibility, regulatory approval, and evidence generation toward routine clinical implementation and broader healthcare impact.

Fetal magnetocardiography remains the reference method for non-invasive assessment of fetal cardiac electrophysiology because it uniquely enables direct evaluation of cardiac rhythm, conduction, and ventricular repolarization. However, widespread clinical adoption has long been constrained by dependence on SQUID technology, which requires cryogenic cooling, dedicated magnetically shielded rooms, and costly infrastructure. The studies included in this review demonstrate that, remarkably, within little more than a decade, OPM-fMCG has evolved from a four-sensor proof-of-concept experiment to clinically evaluated multichannel systems capable of producing signal quality, cardiac interval measurements, and, in selected clinical studies, diagnostic findings comparable to those obtained with conventional SQUID instruments. The principal themes emerging from the available evidence and their relationship to the translational development of OPM-fMCG are summarized in Figure 2.

This translational evolution has been driven by complementary scientific and technological trajectories across several research programs. In the Madison program, which was developed through the work of Ronald Wakai and colleagues, Batie et al. [35] and Strand et al. [36] established the clinical feasibility of OPM-fMCG by demonstrating diagnostic performance comparable to an FDA-cleared SQUID system in both uncomplicated pregnancies and fetuses with clinically significant arrhythmias. In the Little Rock program, led by the late Hari Eswaran and colleagues, Escalona-Vargas et al. [40] focused on engineering optimization, introducing an integrated stand-alone platform, improved acquisition strategies, and systematic evaluation of factors influencing signal quality. In the Munich program, including the work of Peter Fierlinger and colleagues, Wacker-Gussmann et al. [44] reported the implementation of hospital-based OPM-fMCG and measured the first gestational-age-specific reference intervals. Together, these complementary developments illustrate the progressive maturation of OPM-fMCG from experimental technology to clinically feasible diagnostic systems. This technological evolution was also reflected in the transition from custom laboratory-built SERF magnetometers in the earliest studies [33,34] to commercially available dual-axis QuSpin OPMs in almost all subsequent investigations, with the exception of Escalona-Vargas et al. [37], who did not specify the sensor model.

Despite these advances, conventional SQUID-fMCG remains the clinical reference standard. SQUID systems benefit from decades of technical refinement, extensive clinical experience, and well-established reference data, providing a robust benchmark for fetal electrophysiological assessment. By contrast, OPM-fMCG is still in an earlier stage of clinical development, with fewer validation studies, smaller patient cohorts, and more limited experience in routine clinical practice. Rather than replacing SQUID technology immediately, OPMs are progressively narrowing the performance gap. Important technical challenges nevertheless remain. Reliable detection of low-amplitude waveform components, particularly the fetal T wave, continues to be more difficult than identification of the QRS complex, limiting robust assessment of ventricular repolarization in some recordings. Gestational age represents an additional limitation. In contrast to the longer clinical experience with SQUID-fMCG from approximately mid-gestation onward, most OPM-fMCG studies have focused on later gestation, and relatively few observations have been reported before 28 weeks; Wurm et al. [43] included pregnancies from 26 weeks’ gestation, while Wacker-Gussmann et al. [44] focused on 25 weeks’ gestation. Earlier gestational assessment is technically particularly challenging because of the smaller fetal cardiac signal and consequently lower signal-to-noise ratio. Establishing reliable OPM-fMCG performance during the second trimester therefore remains an important translational objective.

Signal quality remains influenced by fetal position, sensor-to-fetus distance, environmental magnetic interference, and methodological differences in sensor configuration and signal processing techniques, resulting in variable detection rates and limiting direct comparison between studies. The reviewed studies employed a variety of signal processing strategies to separate fMCG from maternal cardiac activity and environmental interference, evolving from early eigenvector-based spatial filtering to later ICA- and projection-based approaches. Projection operator algorithms were applied consistently in the Little Rock program, whereas other programs primarily relied on ICA-based approaches, including FastICA and Splined Independent Component Subtraction. Despite these differences in signal separation, preprocessing pipelines were broadly similar, typically comprising band-pass filtering, power line suppression, active magnetic field compensation, ICA-based background noise reduction, or other spatial noise reduction techniques, followed by heartbeat detection and ensemble averaging. This methodological heterogeneity complicates direct comparison between studies and highlights the need for standardized signal processing workflows. A useful precedent for harmonized data structures and reporting standards are developments in the MEG field, such as the Brain Imaging Data Structure extension for MEG (MEG-BIDS) [57] and emerging OPM-FLUX [58]. Both frameworks could be useful for OPM-fMCG.

Beyond technical performance, the translational potential of OPM-fMCG also depends on economic feasibility. Although IASs and other clinically important fetal rhythm disorders carry substantial consequences for affected pregnancies, the absolute number of eligible referrals at an individual center is relatively low. This creates an unfavorable relationship between patient throughput and the high fixed costs of conventional SQUID-fMCG, including the acquisition and maintenance of cryogenic instrumentation and dedicated magnetically shielded facilities. Such costs are difficult to distribute across a sufficiently large number of examinations, helping to explain why SQUID-fMCG has remained concentrated in a small number of highly specialized academic centers despite its demonstrated diagnostic value. OPM technology could potentially change this relationship by eliminating cryogenic cooling and reducing shielding and infrastructure requirements; however, lower technological costs alone would not establish economic viability.

Importantly, broader technical accessibility should not be equated with an expansion from targeted diagnosis to universal prenatal screening. Based on the current evidence, the most plausible near-term clinical role of OPM-fMCG is as a second-line precision diagnostic tool for pregnancies with suspected fetal arrhythmia, abnormal or inconclusive echocardiographic findings, or family history or other risk factors for IASs. Studies in uncomplicated pregnancies have been instrumental for establishing feasibility, signal-quality determinants, physiological measurements, and gestational-age-specific reference intervals, but they do not provide evidence that OPM-fMCG would improve outcomes when applied as a screening test in an unselected obstetric population.

Although advances in non-invasive fetal ECG [59,60,61] have improved fetal heart rate and rhythm assessment, reliable characterization of cardiac conduction intervals and ventricular repolarization remains challenging because electrical signals are substantially attenuated and distorted by maternal tissues and vernix caseosa, maintaining a central role for fMCG in prenatal electrophysiological assessment. Future studies should establish reproducibility across the entire OPM-fMCG workflow, including hardware performance, signal processing algorithms, operator-dependent procedures, and independent validation across centers.

Regulatory approval also remains an important challenge. While OPM-based magnetocardiography systems have already received FDA 510(k) clearance for adult applications [2], no OPM-fMCG system has yet been cleared or approved for fetal use. Similarly, future clinical adoption in Europe will require compliance with the European Medical Device Regulation (MDR), including demonstration of analytical and clinical performance to support CE marking for fetal diagnostic applications.

The published literature now includes more than 200 reported pregnancy-level observations, although some overlap between cohorts is possible. Several Little Rock publications represent sequential development of related OPM-fMCG platforms and used longitudinal recruitment designs. Participant overlap was evident between the two 2020 studies, in which two of the three participants in the initial pilot [37] appeared to be represented in the subsequent twenty-four-participant cohort [38]. For the later prototype [39] and stand-alone system studies [40], the publications do not provide sufficient participant-level identifiers to determine the exact degree of overlap. Accordingly, publication-level enrolment counts should not be summed up to estimate the number of unique pregnancies.

Because OPM-fMCG remains an emerging technology, publication bias toward technically successful reports may have resulted in underrepresentation of unsuccessful prototypes or less favorable findings. Moreover, the methodological quality of the available evidence should be interpreted with caution. Most studies were single-center observational investigations based on relatively small convenience samples, frequently comprising healthy pregnancies or highly selected clinical cases. None were prospective diagnostic-accuracy studies designed to evaluate predefined clinical endpoints, and blinding of signal analysis or outcome assessment was generally not reported. Consequently, the current literature provides encouraging evidence of technical and clinical feasibility but remains susceptible to selection, publication, and observational biases that may overestimate diagnostic performance. Large prospective multicenter diagnostic-accuracy studies are now required to establish the clinical validity, effectiveness, and generalizability of OPM-fMCG. Notably, prospective clinical evaluation of fMCG remains ongoing even for established SQUID systems, as illustrated by the large observational study NCT03047161 (https://clinicaltrials.gov/study/NCT03047161 (accessed on 29 July 2026)). This indicates the need for similarly rigorous prospective evaluation of OPM-based systems. Economic evaluation will also be important to determine whether reduced infrastructure requirements translate into lower overall service costs.

This review has several limitations. Although a comprehensive literature search and predefined eligibility criteria were used, the small and heterogeneous evidence base precluded quantitative synthesis. Considerable variation in study design, participant characteristics, sensor configurations, comparators, and reported outcomes limited direct comparison across studies. No formal study-level risk-of-bias assessment was performed because the evidence consisted predominantly of heterogeneous technical-clinical investigations; methodological limitations were therefore assessed narratively. Furthermore, participant overlap between sequential publications from the same research programs could not always be excluded, potentially affecting the independence of the summarized evidence.

The current evidence base also remains limited in breadth, independence, and clinical maturity. Most studies originate from a small number of closely connected research programs, with little replication across institutions or healthcare settings. Evidence regarding workflow, operator training, repeatability, maternal acceptability, downstream clinical impact, and service costs is also scarce. Addressing these gaps will be essential before widespread clinical adoption can be considered.

Beyond technological development, sustained investment in maternal and fetal cardiovascular research is warranted. Technologies with the potential to reduce fetal mortality and lifelong cardiovascular morbidity deserve continued support. In addition to improving technical performance, the reduced infrastructure requirements of OPM-fMCG systems may facilitate broader access to fetal electrophysiological assessment, particularly in centers where installation of conventional SQUID facilities is impractical. Future translational research should therefore incorporate patient and public involvement together with early health technology assessment to evaluate acceptability, workflow, infrastructure requirements, costs, equitable access, and alignment with clinical priorities alongside diagnostic performance [62,63,64,65].

## 5. Conclusions

Available evidence consistently suggests that OPM-fMCG can achieve signal quality and cardiac interval measurements comparable to those obtained with SQUID systems, together with promising diagnostic concordance in the limited cohorts of fetuses with clinically significant arrhythmias. At the same time, OPM technology eliminates cryogenic cooling and substantially reduces infrastructure requirements, addressing two of the principal barriers to wider clinical adoption of fMCG. Although the evidence base comprises only twelve original studies, they document the translational maturation of OPM-fMCG, from early experimental recordings to multichannel systems evaluated in clinical cohorts. However, prospective multicenter validation, standardized acquisition and signal processing protocols, independent replication, and regulatory approval remain necessary before the technology can be incorporated into routine diagnostic pathways for pregnancies requiring advanced fetal electrophysiological assessment.

## 6. Future Directions

Future research should advance rigorous clinical validation and adoption. Large prospective multicenter diagnostic-accuracy studies should evaluate OPM-fMCG across a broad spectrum of fetal arrhythmias and IASs using predefined reference standards and direct comparison with fetal echocardiography, SQUID-fMCG, and postnatal electrocardiography. Future studies should also specifically evaluate OPM-fMCG at earlier gestational ages, particularly before 28 weeks, and determine the earliest gestational age at which clinically interpretable rhythm, conduction, and repolarization measurements can be obtained reproducibly.

Continued technological development will remain important for advancing OPM-fMCG toward the capabilities of postnatal cardiac imaging and electrophysiology. Ongoing work includes real-time integration of fMCG with simultaneous echocardiographic/Doppler signals, novel assessment of fetal ventricular electromechanical function, and the use of aortic valve clicks for temporal gating when the fetal QRS complex cannot be reliably identified due to a low signal-to-noise ratio. These engineering advances should proceed in parallel with rigorous clinical validation.

Independent replication by centers not involved in technology development will be essential to establish generalizability and reproducibility. International consensus on sensor configuration, patient positioning, acquisition protocols, signal processing, signal-quality metrics, and reporting standards will facilitate comparison between studies, support regulatory evaluation, and accelerate clinical adoption.

Implementation studies should also assess workflow, examination time, operator training, failed or non-diagnostic examinations, maternal acceptability, and overall service costs. These studies should initially focus on clinically defined high-risk and referred populations, in whom the diagnostic value proposition of OPM-fMCG would be strongest; evaluation as a population screening strategy would require separate evidence demonstrating sufficient diagnostic yield, clinical benefit, and cost-effectiveness in unselected pregnancies.

Beyond hardware development, advances in computational analysis are also likely to shape the future evolution of OPM-fMCG. Although not yet established in the included clinical literature, AI-assisted methods may eventually support automated signal-quality assessment, maternal and fetal signal separation, waveform detection, interval measurement, arrhythmia classification, and clinical decision support. However, such approaches will require transparent reporting, clinically representative training datasets, external validation, and safeguards against automation bias before routine clinical use.

Continued investment in this field is justified by the substantial unmet clinical need and the opportunity to improve prenatal diagnosis, reduce preventable fetal mortality, and expand access to advanced fetal cardiac care. Kogutt and Satin [66] compared innovation in obstetrics with several other medical specialties using patent activity and venture-capital investment (VCI) as proxies for technological innovation. Obstetrics ranked lowest, with only 33,913 patents and $1.1 billion in VCI during 2000–2018, and encompassed the fewest funded companies and deals, substantially trailing, for example, orthopedics (378,349 patents and $4.4 billion in VCI), gastroenterology (44,832 patents and $4.8 billion in VCI), dermatology (58,897 patents and $4.9 billion in VCI), and cardiology (1,082,010 patents and $23.4 billion in VCI).

## Figures and Tables

**Table 1:** Articles included in this review that reported the use of fetal magnetocardiography (fMCG) with optically pumped magnetometers (OPMs) up to 14 June 2026 [33,34,35,36,37,38,39,40,41,42,43,44] Participant cohorts may overlap across sequential publications from the same research programs. Numbers represent enrolment reported in each publication and should not be summed as unique pregnancies. For Wacker-Gussmann et al. [44], 57 participants were enrolled and five recordings were excluded because of a low signal-to-noise ratio (SNR) or magnetic artefacts. FHRV—fetal heart rate variability; SQUID—superconducting quantum interference device.

Reference	Year of Publication	Number of Pregnant Women	Population/Clinical Focus	Principal Contribution
**Early studies**				
Wyllie et al. [33]	2012	1	1 healthy pregnancy; normal fetal rhythm at 31 weeks’ gestation	First proof-of-concept OPM-fMCG
Alem et al. [34]	2015	1	1 healthy pregnancy; normal fetal rhythm at 32 weeks’ gestation	Flexible conformal multichannel OPM array
**Clinical studies—Madison program**				
Batie et al. [35]	2018	15	8 uncomplicated pregnancies; 7 pregnancies with fetal arrhythmia or high risk of fetal arrhythmia, including marked QT prolongation/LQTS, functional AV block, ventricular bigeminy or tachycardia, low atrial rhythm, and atrial or ventricular ectopy	First clinical comparison with SQUID
Strand et al. [36]	2019	24	6 uncomplicated pregnancies; 2 pregnancies with high-risk obstetric conditions; 16 pregnancies with fetal arrhythmia or risk of arrhythmia attributed to clinical findings or family history	Compact OPM-fMCG platform
**Clinical studies—Little Rock program**				
Escalona-Vargas et al. [37]	2020	3	3 low-risk uncomplicated pregnancies; normal fetal rhythm	Flexible adaptable sensor array
Escalona-Vargas et al. [38]	2020	24	24 healthy uncomplicated singleton pregnancies, 28–38 weeks’ gestation	Clinical evaluation of flexible arrays for FHRV
Escalona-Vargas et al. [39]	2024	15	15 healthy pregnancies; additional proof-of-concept recording in a fetus with abnormal rhythm	Prototype bed-based system
Escalona-Vargas et al. [40]	2025	22	22 pregnancies undergoing serial paired OPM–SQUID recordings; predominantly low-risk pregnancies with normal fetal rhythm	Stand-alone integrated OPM-fMCG platform
Escalona-Vargas et al. [41]	2025	4	4 low-risk pregnancies, 28–36 weeks’ gestation; fetal movement and heart-rate assessment	Simultaneous fetal movement assessment
Ramirez et al. [42]	2026	32	32 pregnant participants with longitudinal recordings at 28–38 weeks’ gestation; evaluation of maternal and fetal determinants of signal quality and waveform detectability	Determinants of OPM-fMCG signal quality
**Clinical studies—Munich program**				
Wurm et al. [43]	2023	7	7 uncomplicated pregnancies, 26–36 weeks’ gestation; normal fetal rhythm	Hospital implementation
Wacker-Gussmann et al. [44]	2026	57	57 healthy women with uncomplicated singleton pregnancies, 25–40 weeks’ gestation; five recordings excluded because of low SNR or magnetic artefact	First gestational-age-specific reference intervals

**Table 2:** A methodological overview of published OPM-fMCG studies up to 14 June 2026 [33,34,35,36,37,38,39,40,41,42,43,44], summarizing the experimental setups, signal separation methods, preprocessing and noise-reduction techniques, and shielding environment used for fetal cardiac signal extraction and analysis. OPM-fMCG—fetal magnetocardiography (fMCG) with optically pumped magnetometers (OPMs); MSR—magnetically shielded room; OP—orthogonal projection; ICA—independent component analysis; POMN—projection operator based on minimum norm.

Reference	Experimental Setup	fMCG Separation Methods	Preprocessing/Noise Reduction Techniques	Shielding Environment
**Early studies**				
Wyllie et al. [33]	Custom-built SERF OPM array (4 channels; 2 channels closer to the fetus, 2 channels closer to mother’s heart); comparison with a 7-channel vector SQUID magnetometer (21 detectors; Tristan Vector Magnetometer, Tristan Inc., San Diego).	Eigenvector-based spatial filtering to separate fetal and maternal MCG [45].	Active hardware gradiometry (feedback compensation); 80 Hz low-pass filter; 60 Hz comb filter; Autocorrelation-based beat alignment for averaging.	MSR with a noise floor of approximately 5 fT/√Hz
Alem et al. [34]	25-channel microfabricated SERF OPM array mounted on three flexible belts (two over the abdomen, one over the chest); 16 of 25 sensors used for analysis (9 rejected due to excessive noise); the sensors were configured in a software lock-in detection (20 kHz acquisition, 1.7 kHz modulation).	OP using maternal MCG signal-space vectors [46]; ICA using the second-order blind-identification algorithm (SOBI) [47]; simultaneous chest and abdominal recordings used to characterize maternal and fetal MCG.	40 Hz low-pass filter; 0.5 Hz high-pass filter; rejection of noisy channels; QRS detection and autocorrelation/R-peak alignment for averaging.	7-layer BMSR II MSR (remanent field < 1 nT, shielding factor ≈ 10,000 at 0.1 Hz)
**Clinical studies—Madison program**				
Batie et al. [35]	QuSpin QZFM OPM array mounted in a 3D-printed holder (from 3 up to 8 sensors in a 3 × 3 grid with 3.81 cm spacing, center occupied by a support post); comparison with a SQUID magnetometer (Model 624, Tristan Technologies).	Signal processing to remove maternal and environmental interference (method not specified).	Signal processing to remove maternal and environmental interference (method not specified).	2-shell MSR
Strand et al. [36]	10-sensor QuSpin QZFM OPM array (12-slot 3D-printed holder arranged in a 9 × 9 cm offset square grid, with two corner slots left vacant); comparison with a 7-channel vector SQUID gradiometer (21 SQUID sensors; Tristan 624 Biomagnetometer); OPMs measured two orthogonal magnetic field components; subjects were measured prone for OPM-CS and supine (or on one side if necessary) for SQUID-MSR and OPM-MSR.	Maternal MCG was removed using ICA (Splined Independent Component Subtraction—ICS) [48].	Band-pass filtering (1–80 Hz); Linear Minimum Mean-Square Error (LMMSE) spatial filter to attenuate environmental and other interference [49]; autocorrelation-based beat alignment for averaging (50 consecutive beats)	3-shell open-ended cylindrical mu-metal shield (OPM-CS); additional comparison using the same OPM array in a 2-shell mu-metal magnetically shielded room (OPM-MSR) and the SQUID gradiometer in the 2-shell mu-metal MSR (SQUID-MSR); residual DC magnetic field nulled to ≈10 nT using triaxial compensation coils
**Clinical studies—Little Rock program**				
Escalona-Vargas et al. [37]	14-channel OPM array configured using 3D-printed adaptable sensor holders (sensor spacing 3 cm); no details of the OPM model are provided; measurements performed in three maternal positions (leaning backward, leaning forward, prone); fetal heart localized by ultrasound before recordings; 6-min recordings from three healthy pregnant women.	Projection operator algorithm based on minimum norm (POMN) for maternal MCG attenuation and fetal MCG extraction [50].	Band-pass filter (0.5–50 Hz); notch filter (power line attenuation); wavelet transform-based detrending to remove low-frequency baseline drift [51]; R-peak detection using the Hilbert transform [52]; time averaging.	No shielding details reported.
Escalona-Vargas et al. [38]	14-channel QuSpin QZFM Gen-1.0 QZFM OPM array (7 sensors, dual-axis operation); sensors mounted in 3D-printed flexible grid/belt (3 cm sensor spacing) positioned over the maternal abdomen; measurements performed in two maternal positions (leaning forward and leaning backward); fetal heart localized by ultrasound before each recording; 1 kHz sampling rate; two 6-min recordings per session in 24 healthy pregnant women (28–38 weeks GA).	POMN for maternal MCG attenuation and fetal MCG extraction.	Notch filter (power line attenuation); band-pass filter (0.5–50 Hz); principal component analysis (PCA) implemented in the Brainstorm software package (version not specified) for additional noise attenuation; wavelet transform-based detrending; R-peak detection using the Hilbert transform; manual correction of missing/spurious fetal heartbeats; grand averaging	No shielding details reported.
Escalona-Vargas et al. [39]	14 QuSpin OPMs (QZFM Gen-1.0, operated in dual-axis); 3D-printed prototype grid mounted in a customized bed; 3-layer magnetically shielded room (Vakuumschmelze; Hanau, Germany) with active triaxial Helmholtz coils for residual field compensation; prone position with sensors in contact with the maternal abdomen; ultrasound-guided sensor placement; back-to-back comparison with the 151-channel SARA SQUID system.	POMN for maternal MCG attenuation and fetal MCG extraction.	Band-pass filter (0.5–50 Hz); R-peak detection using the Hilbert transform; cubic spline interpolation for uniformly sampled R–R intervals; signal grand averaging	3-layer MSR (Vakuumschmelze; Hanau, Germany) with active triaxial Helmholtz coils for residual field compensation
Escalona-Vargas et al. [40]	14 QuSpin QZFM OPMs (Gen-2 and Gen-3, 28 channels operated in dual-axis mode); customized bed-based stand-alone system with adjustable belly-shaped sensor holder; 1 kHz sampling; subjects measured prone in a “donut-hole” mattress; comparison with the 151-channel SARA SQUID system.	POMN for maternal MCG attenuation and fetal MCG extraction.	Notch filter; 4th-order Butterworth band-pass filter (0.5–50 Hz); ICA (Infomax variant [53], implemented in the Brainstorm) for background noise attenuation; manual rejection of bad channels/segments and ICA component selection; R-peak detection using the Hilbert transform; signal grand averaging	3-layer cylindrical mu-metal shield (3.05 m long × 1.83 m diameter) with passive shielding, degaussing system and active compensation coil; residual field < 15 nT; average noise floor 11 fT/√Hz (10–200 Hz).
Escalona-Vargas et al. [41]	14 QuSpin QZFM OPMs (Gen-2 and Gen-3, 28 channels operated in dual-axis mode); customized bed-based stand-alone system with adjustable belly-shaped sensor holder; 1 kHz sampling; HALO (QuSpin) used for sensor localization; subjects measured prone; ultrasound-guided fetal heart localization.	POMN for maternal MCG attenuation and fetal MCG extraction.	Notch filter; 4th-order Butterworth band-pass filter (1–50 Hz); ICA for background noise attenuation; R-peak detection using the Hilbert transform; visual inspection using Brainstorm.	3-layer cylindrical mu-metal shield (3.05 m long × 1.83 m diameter); average noise floor 11 fT/√Hz (10–200 Hz);
Ramirez et al. [42]	Bed-based stand-alone array of 15 QuSpin QZFM OPMs (30 channels) (Gen-2 and Gen-3, dual-axis operation); custom bed with sensor array positioned directly beneath the maternal abdomen; recordings acquired at 1 kHz; participants measured in the prone position after ultrasound localization of the fetal heart.	POMN for maternal MCG attenuation and fetal MCG extraction.	ICA (Infomax variant, implemented in Brainstorm) used for background noise suppression. Fourth-order Butterworth band-pass filter (0.5–50 Hz); 60 Hz notch filter (1.5 Hz bandwidth); visual inspection and rejection of bad channels/artifacts; R-peak detection; averaging in 60 s windows for cardiac time interval (CTI) extraction.	3-layer cylindrical magnetic shield (Magnetic Shields Ltd.) located inside an MSR shield reduced residual DC magnetic field to < 15 nT.
**Clinical studies—Munich program**				
Wurm et al. [43]	8 QuSpin QZFM OPMs arranged in a 3 × 3 grid (5 cm spacing, center position unoccupied); dual-axis operation; sensors positioned 1.5 mm below the abdomen (8 mm above the sensitive volume); subjects measured in the prone position on a movable bed with an abdominal cutout.	ICA (FastICA implementation [54]) to separate fetal and maternal MCG.	50 Hz notch filter; first-order Butterworth band-pass filter (3–75 Hz); matched filtering based on bispectral analysis [55]; heartbeat detection; averaging (up to 300 consecutive beats) to derive high-resolution fMCG waveforms.	3-layer cylindrical person-sized mu-metal shield (97 cm diameter, 265 cm length) with noise floor ≈ 220 fT/√Hz (3–45 Hz); active triaxial magnetic field compensation reduced the ambient noise floor to ≈80 fT/√Hz.
Wacker-Gussmann et al. [44]	16 QuSpin QZFM OPMs arranged in a 4 × 4 grid (5 cm spacing) below the abdomen; dual-axis operation; measurements performed in a subjects measured prone following ultrasound localization of the fetal heart; three recording runs of at least 10 min each.	ICA with manual maternal component identification. [48]	Digital band-pass filter (1–50 Hz); ICA used for maternal MCG and interference removal; averaging of 50–100 consecutive fetal QRS complexes.	3-layer cylindrical person-sized magnetic shield with active triaxial magnetic field compensation (MR-3, Stefan Mayer Instruments); ambient noise floor ≈ 80 fT/√Hz;

## Data Availability

No new datasets were generated or analyzed during this study. The literature search records supporting the findings of this review are available from the corresponding author upon request.

## Abbreviations

The following abbreviations are used in this manuscript:

ECG	Electrocardiography
FHRV	Fetal heart rate variability
fMCG	Fetal magnetocardiography
IAS	Inherited arrhythmia syndrome
ICA	Independent component analysis
LQTS	Long QT syndrome
MEG	Magnetoencephalography
MSR	Magnetically-shielded room
OP	Orthogonal projection
OPM	Optically pumped magnetometer
POMN	Projection operator based on minimum norm
RMSSD	Root mean square of successive differences
SDNN	Standard deviation of normal-to-normal beat intervals
SNR	Signal-to-noise ratio
SQUID	Superconducting Quantum Interference Device
TdP	Torsades de Pointes
ICS	Splined Independent Component Subtraction

## Appendix A. Structured Literature Search and Review Methods

### Appendix A.1. Research Question and Objective

The objective of this review, based on a comprehensive search and narrative synthesis, was to identify and summarize original human clinical studies evaluating fMCG performed using optically pumped magnetometry or OPMs in pregnant women. This review focused on clinically interpretable fetal cardiac information obtained with OPM-fMCG, including fetal heart rate, fetal rhythm assessment, fetal heart rate variability, cardiac time intervals, diagnostic-quality signal acquisition, comparison with established fetal cardiac assessment methods, maternal tolerability, safety, and evidence of clinical utility where reported. The review excluded studies limited to technical development, phantom testing, simulations, ex vivo specimens, animal experiments, adult-only magnetocardiography, fetal magnetoencephalography without fetal cardiac outcomes, and non-original publications. The review question was “What clinically relevant fetal cardiac information is reported from fetal magnetocardiography performed using optically pumped magnetometry or optically pumped magnetometers in pregnant women, based on original in vivo human studies?” and was addressed with reference to the PICOS criteria listed in Table A1.

**Table A1:** PICOS criteria addressing the research questions.

PICOS	Included	Excluded
**Population**	Pregnant women or pregnant people carrying a fetus of any gestational age; normal pregnancies or pregnancies with suspected or known fetal cardiac rhythm or conduction abnormalities.	Non-human studies; animal studies; cell lines; phantoms; ex vivo tissue only; adult-only or neonatal-only populations without fetal in vivo pregnancy data.
**Intervention**	Fetal magnetocardiography performed using optically pumped magnetometry, optically pumped magnetometers, optical magnetometers, atomic magnetometers, SERF magnetometers, or closely equivalent OPM-based fetal cardiac magnetic recording systems.	SQUID-only fetal magnetocardiography; fetal echocardiography, CTG, fetal ECG, or postnatal ECG without OPM-based fetal magnetocardiography; fetal magnetoencephalography without fetal cardiac outcomes.
**Comparator**	No comparator required	Comparator-only studies without OPM-based fetal magnetocardiography.
**Outcomes**	Clinically interpretable fetal cardiac outcomes, including fetal heart rate, fetal heart rate variability, rhythm assessment, arrhythmia or conduction assessment, P/PR/QRS/QT/QTc/RR intervals, waveform detectability, diagnostic-quality signal acquisition, agreement with clinical comparators, maternal tolerability, safety, or clinical utility.	Purely technical outcomes only, such as sensor noise, shielding performance, algorithm performance, simulation accuracy, or phantom validation, without in vivo pregnant-human fetal cardiac data.
**Study design**	Original in vivo human studies, including prospective or retrospective clinical studies, feasibility studies, diagnostic or comparative studies, cohorts, case series, and clinically informative case reports.	Reviews, systematic reviews, meta-analyses, editorials, comments, letters, protocols, conference abstracts without sufficient original clinical data (but full peer-reviewed conference proceedings containing adequate original data were eligible), educational articles, methodological-only papers, and purely technical development reports.

### Appendix A.2. Search Strategy

To provide a transparent and reproducible account of literature identification, the database-specific search strategies are reported in full below. Searches were conducted on 14 June 2026, and the corresponding numbers of records retrieved are presented in Table A2, Table A3 and Table A4.

**Table A2:** Search strategy and number of hits in PubMed.

PubMed	14 June 2026	
#1 (Population)	(fMCG[tiab] OR fetal MCG[tiab] OR “foetal MCG”[tiab] OR ((fetal[tiab] OR foetal[tiab] OR fetus[tiab] OR foetus[tiab]) AND magnetocardiograph*[tiab]))	1741
#2 (Intervention)	(OPM[tiab] OR OPMs[tiab] OR “optically pumped”[tiab] OR ((optical[tiab] OR atomic[tiab]) AND magnetometer*[tiab]) OR SERF[tiab] OR “spin-exchange relaxation-free”[tiab] OR “spin exchange relaxation free”[tiab] OR QuSpin[tiab])	5135
#1 AND #2		21

* denotes a wildcard representing any continuation of the preceding word stem.

**Table A3:** Search strategy and number of hits in Embase.

Embase	14 June 2026	
#1 (Population)	(‘magnetocardiography’/exp OR fMCG:ti,ab,kw OR ‘fetal MCG’:ti,ab,kw OR ((fetal:ti,ab,kw OR foetal:ti,ab,kw OR fetus:ti,ab,kw OR foetus:ti,ab,kw OR pregnan*:ti,ab,kw OR gestation*:ti,ab,kw OR prenatal:ti,ab,kw OR antenatal:ti,ab,kw) AND (magnetocardiograph*:ti,ab,kw OR magnetocardiogram*:ti,ab,kw OR MCG:ti,ab,kw OR ‘cardiac magnetic field*’:ti,ab,kw)))	4237
#2 (Intervention)	(OPM:ti,ab,kw OR OPMs:ti,ab,kw OR ‘optically pumped’:ti,ab,kw OR ‘optical pumping’:ti,ab,kw OR ((optical:ti,ab,kw OR atomic:ti,ab,kw OR alkali:ti,ab,kw OR quantum:ti,ab,kw) AND magnetometr*:ti,ab,kw) OR ‘magnetic sensor*’:ti,ab,kw OR SERF:ti,ab,kw OR ‘spin-exchange relaxation-free’:ti,ab,kw OR ‘spin exchange relaxation free’:ti,ab,kw OR QuSpin:ti,ab,kw)	4845
#1 AND #2		89
#1 AND #2	filter: Article, Article in press, Conference abstracts	72

* denotes a wildcard representing any continuation of the preceding word stem.

**Table A4:** Search strategy and number of hits in Scopus.

Scopus	14 June 2026	
#1 (Population)	TITLE-ABS-KEY(fMCG OR “fetal MCG” OR ((fetal OR foetal OR fetus OR foetus OR pregnan* OR gestation* OR prenatal OR antenatal) AND (magnetocardiograph* OR magnetocardiogram* OR MCG OR “cardiac magnetic field*”)))	3986
#2 (Intervention)	TITLE-ABS-KEY(OPM OR OPMs OR “optically pumped” OR “optical pumping” OR ((optical OR atomic OR alkali OR quantum) AND magnetometr*) OR “magnetic sensor*” OR SERF OR “spin-exchange relaxation-free” OR “spin exchange relaxation free” OR QuSpin)	73,849
#1 AND #2		32
#1 AND #2	filter: Article, Conference paper	24

* denotes a wildcard representing any continuation of the preceding word stem.

### Appendix A.3. Exclusion Criteria

Records were excluded if they met any of the following criteria (hierarchical):

1. No title/abstract available or not in English.
2. Not an original study.
3. Non-human, animal, phantom, simulation, ex vivo-only, or tissue-only study.
4. Not an in vivo pregnant-human fetal study.
5. OPM-based fetal magnetocardiography not used.
6. No clinically interpretable fetal cardiac outcome reported (purely technical, hardware, sensor, shielding, algorithmic, or methodological study without original pregnant-human fetal cardiac data).
7. Ineligible publication type, including review, systematic review, meta-analysis, editorial, commentary, letter, protocol, conference abstract, or educational article.

### Appendix A.4. Additional Searches

To ensure that no recently published or poorly indexed studies were missed, a brief, targeted supplementary search was performed after the primary database searches, which resulted in one additional record [42].
